# Multiple polyurethane implant punctures during fat grafting: case report and review of the literature

**DOI:** 10.1186/s12893-020-00915-4

**Published:** 2020-10-20

**Authors:** Dmitry Batiukov, V. Podgaiski, D. Mikulich, S. Kalinin

**Affiliations:** 1 Medical Center “Antes Med”, Kozlova Lane 25-7, 220037 Minsk, Belarus; 2 Belorussian Medical Academy of Postgraduate Education, Brovki 3, b. 3, 220013 Minsk, Belarus; 3 Breast Cancer and Reconstructive Surgery Department, National Cancer Centre of Belarus, Minsk District, 223040 Lesnoy, Minsk Region Belarus; 4 Department of Plastic and Aesthetic Surgery of the 4th City Clinical Hospital Named After N.E. Savchenko, Rozy Lyuksemburg str. 110, 220089 Minsk, Belarus

**Keywords:** Polyurethane implants, Fat grafting, Implant puncture, Breast reconstruction, Breast augmentation, Implant puncture

## Abstract

**Background:**

Breast augmentation with implants continues to be the most popular aesthetic surgical procedure performed worldwide. Fat grafting may improve the results of breast augmentation and breast reconstruction with implants. However, fat grafting to the breast with implants carries the risk of implant puncture. To our best knowledge this is the first case in which polyurethane implant puncture during fat grafting is described.

**Case presentation:**

We report multiple bilateral implant punctures with the cannula during fat grafting in a patient who previously underwent breast reconstruction with polyurethane implants.

**Conclusions:**

Implants that promote tissue ingrowth may be more prone to puncture with the cannula during fat grafting. Specific planning and surgical maneuvers decrease the risk of implant puncture.

**Level of evidence:**

Level V, case report.

## Background

Breast augmentation (BA) with implants continues to be the most popular aesthetic surgical procedure performed worldwide [1, 2]. Fat grafting (FG) may improve the results of BA and breast reconstruction with implants by thickening and refining the skin envelope, filling residual defects (rippling, double bubble etc.), adding volume, changing the form and avoiding submuscular implant placement [3–13]. Simultaneous combination of BA with implants and FG is called hybrid or composite BA [6, 11, 12, 14, 15]. FG to the breast with implants inside carries the risk of the implant puncture. This is particularly possible if FG is performed as a secondary procedure without visualizing the implant pocket, when tissues are thin, fibrous and force is needed to pass the cannula. The incidence of implant puncture during fat grafting is not reported in the literature.

## Case presentation

**Fig. 1:**
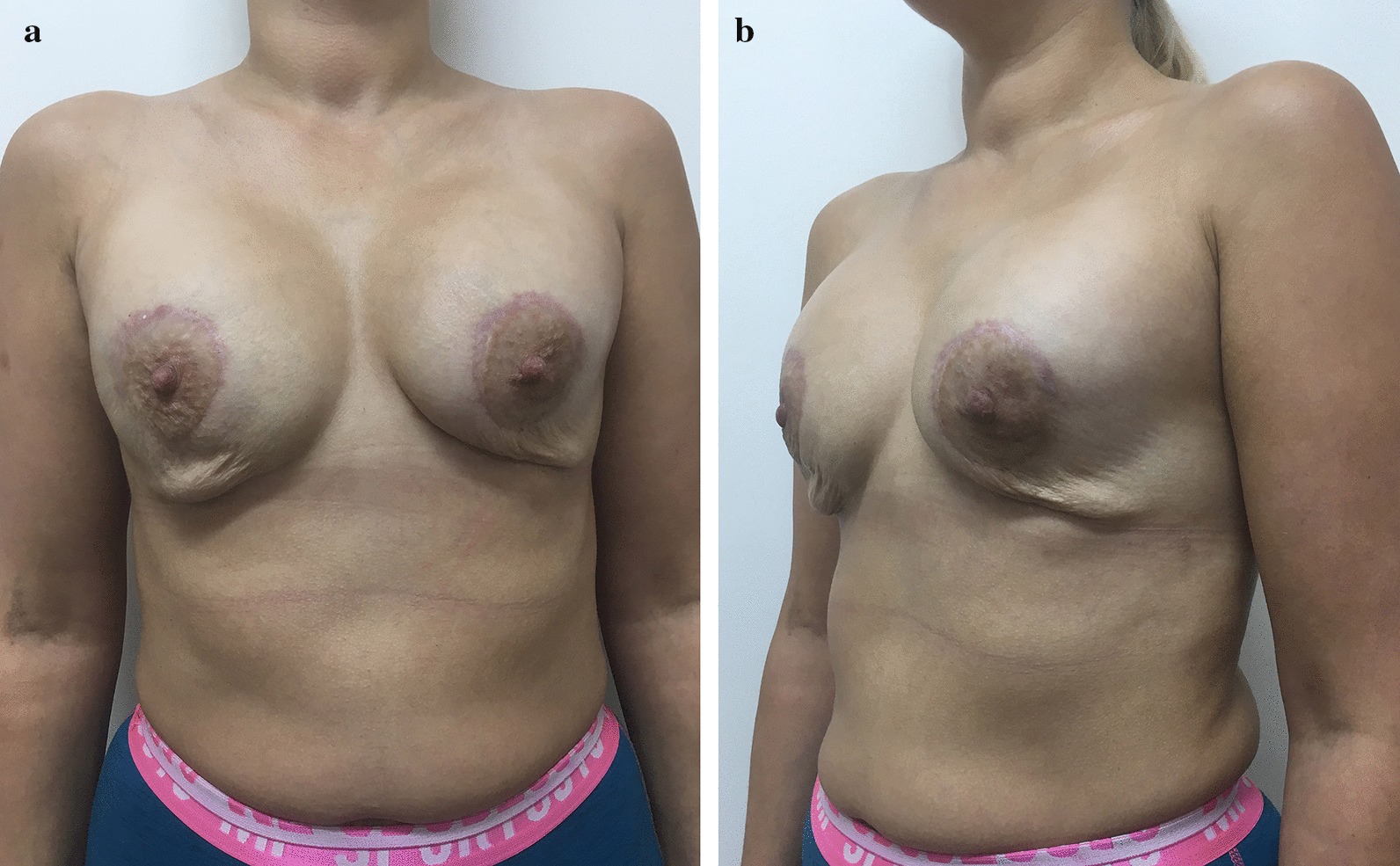
Patient B 8 months after DTI reconstruction with polyurethane implants and periareolar mastopexy, 4 months after secondary periareolar mastopexy. High riding implants. Thin envelope, particularly in the lower pole. **a** Frontal view, **b** oblique view

**Fig. 2:**
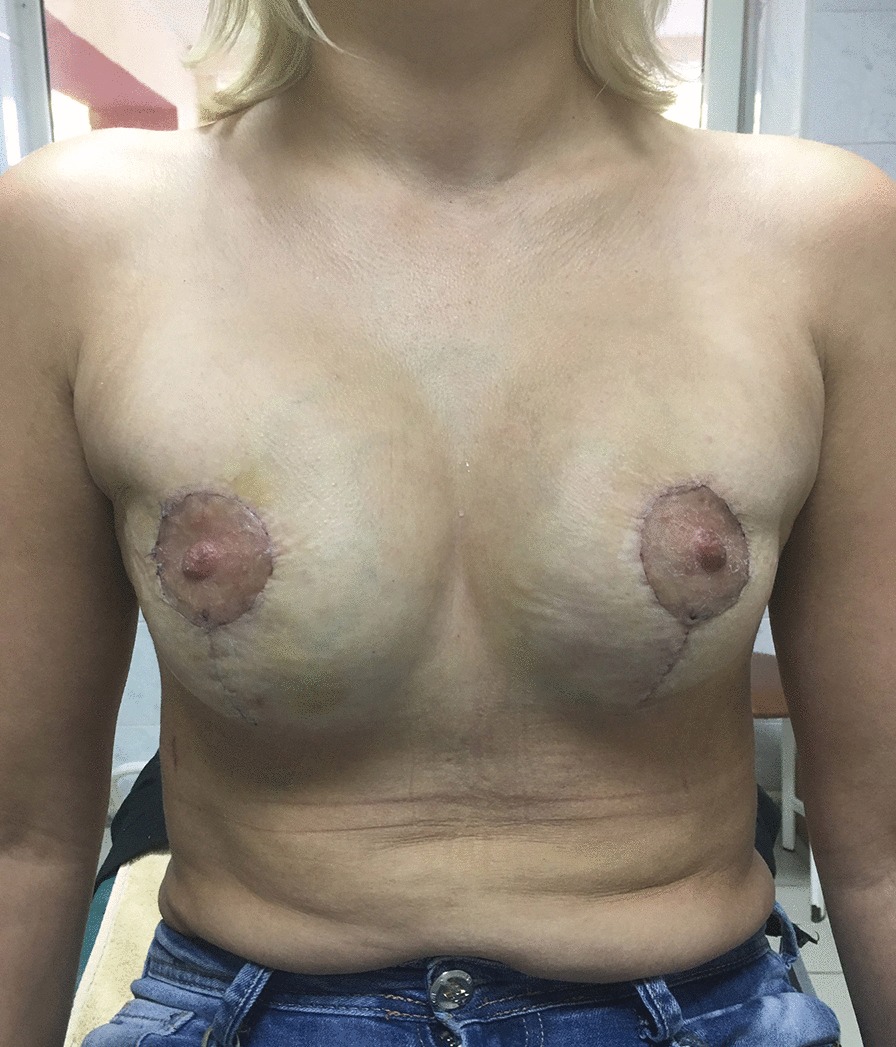
Patient B 3 weeks after implant exchange for new polyurethane implants, capsulectomy and circumvertical mastopexy

**Fig. 3:**
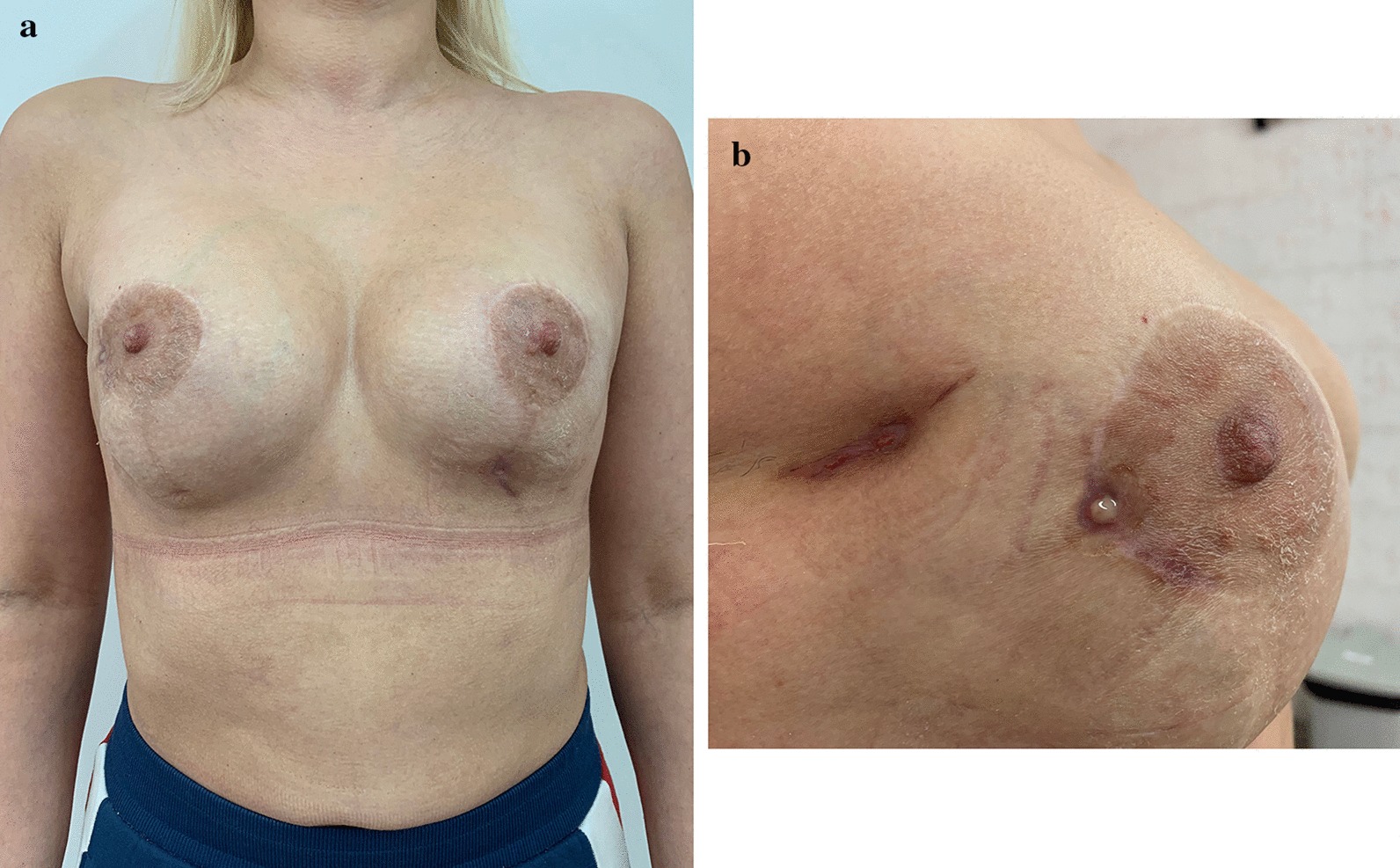
Patient B 4 weeks after FG bilaterally. **a** Frontal view, **b** right breast, lateral view

Patient B, 36 years old made an appointment at another institution complaining of multiple painful lumps in both breasts. She already had a sectoral resection of the right breast because of multiple fibroadenomas two years ago. The anamnesis was unremarkable. She was diagnosed with bilateral fibrocystic breast disease, multiple fibroadenomas of the right breast, BRCA negative. Bilateral subcutaneous mastectomy with DTI subpectoral reconstruction with polyurethane implants and periareolar mastopexy was performed. The postoperative period was unremarkable. Three months after the surgery on the follow up the patient complained of the breast asymmetry and soft tissue deformity and 4 months after primary surgery secondary periareolar mastopexy was performed. The postoperative course was uneventful. 4 months after the second surgery the patient showed up again with the complaints on the breast asymmetry, contour deformity and skin redundancy of the lower pole (Fig. 1a, b). Secondary BA with capsulectomy, implant exchange for new polyurethane implants and circumvertical mastopexy were performed. The postoperative course was uneventful. But soft tissue deficit of the lower pole persisted (Fig. 2). One month after the last surgery FG to the breast was performed (app. 200 ml per breast). 30 days later the patient complained on the local tender nodules in breast bilaterally, redness of the skin. No hyperthermia was determined. Ultrasound revealed implants to be intact, multiple nodules of the lower and lateral quadrants containing fluid were found. Draining of several nodules was performed and antibiotic therapy started. The treatment was intermittently continued for 3 months. Then the patient made an appointment in our clinic (Fig. 3a, b). Implant rupture was suspected in MRI and implant removal with capsulectomy was done. Multiple punctures of both implants were revealed during surgery (Additional file 1, Video 1). The wounds healed primarily. The patient refused to undergo further surgeries.

## Discussion and conclusions

In 1895 Czerny introduced fat transplantation to the breast [16]. Bruning, Fisher and Illouz were the first to develop liposuction and FG [17–19]. FG was then introduced to the breast, its safety proven and nowadays FG to the breast has become a routine procedure with various indications [20–30].

The literature on the possible implant injury during FG is scarce [31–35]. The authors mainly discuss the advantages of FG and usual complications as non-predictable graft take, irregularities, infection, cysts, fat necrosis [30, 36–38]. Pneumothorax as a result of FG that has similar mechanism as implant puncture is mentioned in some articles [30, 38].

FG and implant placement can be done simultaneously or in several stages. In case of simultaneous surgery, the surgeon can perform FG on the old implant or using a sizer, introducing the implant after FG or protecting the implant and visualizing its integrity before the wound closure if FG is performed after implant placement [6, 35, 39]. Grafting after the implant placement has the advantage of precise shaping and volume control [40, 41]. It also reduces traumatization of fat and thus potentially adds to fat survival.

If FG is performed after wound closure or at the second stage, it is difficult to control the implant integrity. Recently ultrasound was proposed to intraoperatively control gluteal FG [42]. Although the authors do not have this experience, we consider it to be beneficial in compromised areas to establish proper plane of grafting.

Several measures are important to prevent implant puncture and its consequences.

Detailed informed consent in which a patient agrees to accept the risk of implant puncture, as well as the costs involved in corrective surgery should be signed [33]. Aging of the implants resulting in reduction in tensile strength, tear strength, and elongation of the shell may end with rupture or be the contributing factor of incidental implant puncture during FG [43–45]. Thus, MRI should be done preoperatively and the surgeon should consider feasibility of implant exchange in some cases. Optimistically MRI should be performed also postoperatively to rule out implant puncture during FG [33].

To avoid implant injury some authors do not perform FG on the implants with tight, thin breast pockets and scar tissues [33]. Not only does preinfiltration add to the vasoconstriction and anesthesia but it also expands the tissues, which facilitates FG in these problematic areas [40, 41, 46]. FG with the needle is also reported to be useful in scar tissue with very thin envelope over the implant [31].

However, FG to the breast is usually recommended to perform with cannulas [47]. Cannula characteristics influence the possibility of implant puncture. The greater the diameter of the cannula the less likely it may penetrate the implant, and the tip of the cannula can be controlled with more precision. Rigid bent cannulas may also prevent inadvertent implant puncture and help to stay superficially. However, big diameter of the cannula can considerably complicate its advancement in tissues and may interfere with graft survival due to substantial volumes of fat injected in the same spot. The longer the cannula itself and the longer the part of the cannula inside the body, the less the surgeon controls the position of the tip, thus increasing the possibility of inadvertent implant puncture. Obviously, blunt tipped cannulas are less capable of implant puncturing [47]. It has also been also shown that if the holes at the tip of the cannula are positioned at the end (not on the side) significantly less force is needed to penetrate the implant [34].

Technical details of FG directly affect the possibility of implant puncture. FG should be performed subcutaneously [6]. We recommend to use multiple injection points and multiple passes not only to distribute the fat evenly but also to reach recipient areas easier which may contradict the experience of other surgeons who advise to avoid multiple passes in areas with less protection [35]. Longitudinal injection technique also may help to avoid deep injection [33]. Remaining breast implant capsule may protect the implant during FG. It also facilitates FG itself preventing fat from seeping into implant pocket. However, if capsulectomy is planned after FG extra caution is required not to leave free fat in the implant pocket.

Cannula displacement rate during FG directly correlates with the possibility of implant rupture. The more slowly the surgeon moves the cannula the more force he or she needs to rupture the implant [34]. Some authors recommend the ‘sweep technique’ which means that the cannula does not move freely from left to right being in subcutaneous tissues (as opposed to the implant pocket) [35]. However, if the implant is already punctured by the cannula the movements of the cannula from left to right will be also constrained. Thus, FG of the implant may occur. The other factor is that with polyurethane implants there exist no such free space at all because of tissue ingrowth. So, implant type should be known not to be mistaken.

Manipulating the tissues with nondominant hand while advancing the cannula (pinching the skin, lifting it from the implant, distracting etc.) facilitates cannula advancement in a proper direction [33].

Smooth and textured implants move freely inside the pocket. Thus, implant displacement to avoid inadvertent implant puncture is suggested [33, 48]. However, with polyurethane implants this maneuver is useless and even harmful because the tissues are stuck to the implant. In the described case FG was performed one month after polyurethane implant placement when tissue ingrowth may have already become substantial [49]. This probability along with thin tissues and scaring played an important role in the implant puncture. Adherence properties of the implants were mentioned in one publication as a risk factor thus, the authors recommended an open procedure, with or without FG as a safer option [33]. However, FG seems unavoidable in many cases and scars are always the last option. Lastly, implant displacement maneuver may also lead to fat injection into implant pocket that will create another problem (fat necrosis). Thus, we consider that staying superficial with the cannula with the tip facing the skin is more reliable to avoid these two complications.

In summary, the following recommendations diminish the risk of puncture:

- Consider performing FG without the implant in place, on the old implant before implant exchange or on the expander or sizer;
- If the implant is placed simultaneously, perform FG before wound closure;
- If FG is performed secondarily, obtain the data about the implants used for the patient and their biointegration properties. Tissue ingrowth may contribute to implant puncture;
- Be aware of old implants that may have lost elasticity and strength of the shell—consider implant exchange with simultaneous FG;
- Consider infiltration for hydrodissection and vasoconstriction; use a needle for very superficial injections in thin tissues;
- Use ultrasound intraoperatively to guide the cannula and establish the proper plane of grafting in compromised areas;
- Consider performing pretunelling;
- Use blunt tipped cannulas with side-positioned holes;
- Direct the tip of the cannula towards the skin surface, use bended cannulas;
- Inject longitudinally;
- Move the cannula slowly and gently;
- Manipulate the soft tissues with nondominant hand while advancing the cannula;
- Choose multiple injection entry points to graft difficult and remote areas;
- Use deeper positioning of the implant (retrofascial, retropectoral);
- Displace the implant away from the injection site;
- Avoid intrapectoral FG.

## Supplementary information

**Additional file 1: Video 1.** Multiple right breast implant punctures found with the implant in situ.

## Data Availability

Data sharing is not applicable to this article as no datasets were generated or analysed during the current study.

## Abbreviations

BA: Breast augmentation
FG: Fat grafting
MRI: Magnetic resonance imaging
BRCA: Breast cancer gene
DTI: Direct to implant

## Human and animal participants

This article does not contain any studies with human participants or animals performed by any of the authors.
